# Selected Rhizosphere Bacteria Help Tomato Plants Cope with Combined Phosphorus and Salt Stresses

**DOI:** 10.3390/microorganisms8111844

**Published:** 2020-11-23

**Authors:** Gylaine Vanissa Tchuisseu Tchakounté, Beatrice Berger, Sascha Patz, Matthias Becker, Henri Fankem, Victor Désiré Taffouo, Silke Ruppel

**Affiliations:** 1 Leibniz Institute of Vegetable and Ornamental Crops, Theodor-Echtermeyer-Weg 1, 14979 Grossbeeren, Germany; ruppel@igzev.de; 2 Department of Plant Biology, Faculty of Sciences, University of Douala, 24157 Douala, Cameroon; fankemhenri@yahoo.fr (H.F.); dtaffouo@yahoo.com (V.D.T.); 3 Faculty of Life Sciences Humboldt-University of Berlin, Invalidenstraße 42, 10115 Berlin, Germany; 4 Institute for National and International Plant Health, Julius Kuehn-Institute–Federal Research Centre for Cultivated Plants, Messeweg 11/12, 38104 Braunschweig, Germany; beatrice.berger@julius-kuehn.de (B.B.); matthias.becker@julius-kuehn.de (M.B.); 5 Algorithms in Bioinformatics, Center for Bioinformatics, University of Tuebingen, Sand 14, 72076 Tuebingen, Germany; sascha.patz@uni-tuebingen.de

**Keywords:** bacterial inoculum, plant growth, phosphate solubilization, salinity tolerance, combined stress, motility

## Abstract

Plants are often challenged by multiple abiotic stresses simultaneously. The inoculation of beneficial bacteria is known to enhance plant growth under these stresses, such as phosphorus starvation or salt stress. Here, for the first time, we assessed the efficiency of selected beneficial bacterial strains in improving tomato plant growth to better cope with double stresses in salty and P-deficient soil conditions. Six strains of *Arthrobacter* and *Bacillus* with different reservoirs of plant growth-promoting traits were tested in vitro for their abilities to tolerate 2–16% (*w/v*) NaCl concentrations, and shown to retain their motility and phosphate-solubilizing capacity under salt stress conditions. Whether these selected bacteria promote tomato plant growth under combined P and salt stresses was investigated in greenhouse experiments. Bacterial isolates from Cameroonian soils mobilized P from different phosphate sources in shaking culture under both non-saline and saline conditions. They also enhanced plant growth in P-deficient and salt-affected soils by 47–115%, and their PGP effect was even increased in higher salt stress conditions. The results provide valuable information for prospective production of effective bio-fertilizers based on the combined application of local rock phosphate and halotolerant phosphate-solubilizing bacteria. This constitutes a promising strategy to improve plant growth in P-deficient and salt-affected soils.

## 1. Introduction

An ever increasing human population, especially in developing countries, leads to the pressing needs to provide food security to upcoming generations [1]. Therefore, improving yield and sustaining soil fertility are of major interest to agricultural production worldwide. Phosphorus (P) depletion is a major factor in limiting plant growth and crop yields. P is essential in plants for energy production, photosynthesis, proper plant maturation, and stress mitigation. Although P compounds are abundant in soils, the majority occurs in an insoluble form. Consequently, on about 30–40% of the world’s arable land crop, productivity is limited by P deficiency [2]. Usually, P deficiency is addressed by the application of soluble phosphate fertilizers [3]. However, about 60–70% of phosphate fertilizers applied are either adsorbed to iron, aluminum oxides, or calcium, and are no longer directly available to the plant [4]. This problem is exacerbated by salinity, which is increasing in many agricultural soils, especially in semi-arid and arid regions, where agriculture performs under irrigation [5]. Salinity negatively affects almost all aspects of plant development, including germination, vegetative growth, and reproductive stages. It also suppresses P uptake via plant roots [6]. P fixation in soils requires frequent applications of P nutrients. However, expensive phosphate fertilizers represent a major outlay for resource-poor farmers in developing countries like Cameroon.

P availability can also be enhanced by applying plant growth-promoting rhizobacteria (PGPR) that exhibit phosphate-solubilizing activities as bio-inoculants. Different rhizosphere-colonizing bacteria have been demonstrated to release organic phosphates or to solubilize inorganic phosphate compounds, such as di-and tricalcium phosphates, hydroxyapatite, and rock phosphates (RPs) [7]. Nevertheless, the phosphate-solubilizing activity of PGPR is often decreased in saline soils [8]. The ability of bacteria to adapt to P deficiency in salty soils is therefore essential for their growth, survival, and propagation. Bacteria survival also depends on their motility capacity, a common trait among bacteria that confers a persistence advantage in stressed environments. Motility allows bacteria to escape local stresses, translocate to a better nutritional condition in competitive environments, and efficiently invade host tissue. In fact, bacterial surface motility is emerging as a major trait involved in many functions of plant-associated bacteria in regard to their ability to colonize and spread on/in their hosts [9]. Thus, seeking phosphate-solubilizing bacteria (PSB) possessing traits, such as salt tolerance and high motility, may open new perspectives for improving crop productivity in P- deficient and salt-affected soils.

Members of the genera *Arthrobacter* and *Bacillus* represent some of the more important soil bacteria, and are among PGPRs possessing many other PGP traits, such as atmospheric nitrogen fixation, phytohormone synthesis, production of siderophores, and plant pathogen suppression, besides their salt tolerance and phosphate-solubilizing abilities [10]. Merging of multiple PGP traits in bacterial strains is expected to be advantageous for plant development under multiple types of adverse conditions. Several studies have underlined that stimulated plant growth by PGPR is rarely caused by a single bacterial activity but is the net result of various mechanisms of action that are triggered simultaneously. Thus, bacteria used in vivo should ideally exhibit different growth promotion traits for a greater effect. Increased plant growth and nutrient uptake have been reported in many crop species as a result of PGPR application under P starvation [7,11,12] or saline conditions [10,13]. However, little research has been published on bacterial performance on plants exposed to concomitant P deficiency and salt stress. Recent studies reported the beneficial effect of PGPR inoculation on peanut [4] and maize [14] plant growth in the presence of P and salt stresses. However, Mittler [15] stated that a combination of stresses should be regarded as a new state of abiotic stress, since the response of each plant to bacterial inoculation under specific conditions is unique.

Therefore, we investigated the effect of six *Arthrobacter* and *Bacillus* strains carrying different sets of PGP traits with importance for growth improvement and P uptake, on tomato plants under P starvation combined with different levels of salt stress. We assumed that bacteria able to solubilize P compounds in vitro even under saline conditions and possessing multiple PGP functional traits will better improve tomato plant growth and P uptake, compared to strains of the same genera possessing fewer PGP traits. Our main goals were to: (i) valuate the ability of the strains to solubilize various P sources in vitro; (ii) test in vitro the six bacterial strains for their ability to tolerate increasing NaCl concentrations, while retaining their motility; (iii) determine the bacterial phosphate-solubilizing activity under normal and salt stress conditions; and (iv) assess the bacterial inoculation effect on growth and P uptake of tomato plants under P stress combined with different levels of salt in a greenhouse.

## 2. Materials and Methods

### 2.1. Microorganism Selection and Storage

Three *Arthrobacter* strains (V54, V64, and V84) and three *Bacillus* strains (V62, V39, and V1) isolated from maize rhizosphere were used in this study. Bacterial strains were identified based on their partial 16S rRNA gene sequence as recently reported [16]. The partial 16S rRNA gene sequences of the bacterial strains were deposited in the NCBI data base under the accession numbers: MN128890-MN128895. Strains were stored at −80 °C in standard nutrient broth (Carl Roth, Karlsruhe, Germany) medium with glycerol (50% *v/v*). The six strains were selected from a collection of 143 strains due to their different functional abilities when tested for their potential to tolerate salt, to solubilize seven compounds of inorganic phosphate and to fix atmospheric nitrogen (by the presence of the *nif*H gene) (Table S1) [16].

### 2.2. Bacterial Inoculum Preparation

To prepare an inoculum from each bacterial strain, a single pure bacterial colony was transferred into a 100-mL Erlenmeyer flask containing 50 mL of nutrient broth and grown on a rotary shaker at 28 °C for 24 h. Bacterial cells were harvested and washed three times in 0.05 M sterilized NaCl solution after centrifugation (10,000× *g*) for 15 min at 4 °C and finally suspended in 0.05 M sterilized NaCl solution. The optical density (OD) of the suspension was adjusted using an Anthos htlll spectrophotometer (Anthos Mikrosysteme GmbH, Friesoythe, Germany), to 0.2 at 620 nm wavelength, which meant that the population reached 10^8^ colony forming units (CFU) mL^−1^, to be used in greenhouse experiments as described in Section 2.5. This bacterial concentration was proven to be optimal in previous experiments.

### 2.3. Characterization of Bacterial Strains under Normal Condition

#### 2.3.1. Motility Test

To assess the swarming ability of the bacterial strains, 10 µL of each bacterial suspension adjusted to OD 0.2 at 620 nm were spotted at the center of a plate containing 25 mL of a semisolid nutrient broth medium (NB; per liter: 5 g peptone, 3 g yeast extract, and 5 g of Agar-Agar; Carl Roth, Karlsruhe, Germany). For the swimming motility assay, the plate containing the nutrient broth medium (NB; per liter: 5 g peptone, 3 g yeast extract, and 3 g of Agar-Agar) were inoculated with 10 µL of each bacterial suspension on the surface of the plate. The plates in triplicate were incubated at 28 °C for 48 h and motility (diameter of migration of the bacteria on top of the agar) was measured from the center towards the periphery of the plate.

#### 2.3.2. Qualitative Characterizations of Bacteria for Phosphate Solubilization on Plates

The efficacy of the different strains to solubilize sparingly soluble phosphate sources was first assessed on plates. The ability of the bacterial strains to solubilize all seven different inorganic phosphate sources: tricalcium phosphate (TCP), hydroxyapatite, and five rock phosphates (RPs) of different origins (Algeria, Cameroon, Mali, Mexico, and Morocco) was assessed on plates filled with the National Botanical Research Institute’s Phosphate growth medium (NBRIP; [17]). All RPs were analyzed for their chemical contents (Table S2). In total, 10 µL of each bacterial suspension were transferred onto a single point of compartmented Petri dishes. Plates in triplicate for each treatment and each phosphate source were incubated at 28 °C for 5 days. The halo (yellow) zone surrounding the bacterial colony indicated phosphate solubilization. The solubilization index (SI) was used as an indicator for the isolate´s efficiency, and was calculated in the following way: SI = (colony diameter + diameter of halo zone)/colony diameter [18].

#### 2.3.3. Quantitative Estimation of Phosphate Solubilization in Liquid Broth

Bacterial strains were tested in liquid NBRIP media to assess their capability to release P from TCP and Cameroonian RP (CRP). The local RP source (CRP) as well as TCP, the most used inorganic phosphate, were chosen for screening phosphate-solubilizing microorganisms. In all cases, 50 mL of NBRIP medium were distributed into 100-mL Erlenmeyer flasks. After sterilization and cooling, 1 mL of the bacterial suspensions was used to inoculate flasks. Each treatment was replicated three times and non-inoculated flasks supplemented with different phosphate sources and 1 mL of 0.05 M sterile NaCl served as controls. Incubation was performed at 28 °C, 180 rpm for 5 days. At the end of the incubation time, the cultures were transferred into sterile falcon tubes, centrifuged at 10,000× *g* for 10 min at 4 °C, and the supernatants filtered through 0.2-µm filters. The pH of the filtrate was measured in each case using a pH 110 meter (VWR, Darmstadt, Germany). and the available P was determined following the colorimetric molybdate blue method described by [19].

### 2.4. Characterization of Bacterial Strains under Salt Stress

#### 2.4.1. Bacterial Tolerance to Salt

Salt tolerance of bacterial strains was tested on Standard I Nutrient agar (Carl Roth, Karlsruhe, Germany) plates amended with various concentrations of NaCl (2–16%; *w/v*) as described by [10].

#### 2.4.2. Motility Test under Salt Stress

The swarming and swimming ability of bacterial strains under salt stress was performed essentially as described above, on the same media supplemented with different concentrations of NaCl (2–16%; *w/v*), and motility was measured as before.

#### 2.4.3. Phosphate Solubilization under Salt Stress

The influence of salt on the phosphate-solubilizing ability of the bacterial strains was performed essentially as described above, on NBRIP medium supplemented with different concentrations of NaCl (2 and 4%, *w/v*).

### 2.5. Plant Inoculation Experiment

A greenhouse experiment using tomato plant (*Solanum lycopersicum* L., cultivar *Harzfeuer F1*, 94% germination rate) was conducted to evaluate the inoculation effect of the six bacterial strains: (*Arthrobacter* strains V54, V64, and V84; *Bacillus* strains V62, V39, and V1) on plant growth and P uptake under different growth conditions in the greenhouse. Phosphorus stress was triggered by fertilization with poorly soluble Cameroonian RP (CRP). The growth conditions included three treatments: P stress without salt stress (CRP + EC = 0 ds m^−1^), P stress + low salt stress (CRP + low S; CRP + EC = 8 ds m^−1^), and P stress + high salt stress (CRP + high S; CRP + EC = 12 ds m^−1^). The inoculation comprised eight treatments, including six bacterial treatments (inoculated plants with each one of the bacterial strains), a non-inoculated control (negative control), and a non-inoculated treatment fertilized with KH_2_PO_4_ (without phosphorus stress). Surface-sterilized tomato seeds were inoculated by immersion in 1 mL of each bacterial suspension (OD = 0.2 at 620 nm) (microbial treatments) or 1 mL 0.05 M sterilized NaCl solution (control treatments) for 15 min, then sown in quartz sand and maintained in a phyto-chamber (25/20 °C day/night temperature) for 14 days. Afterwards, seedlings were potted in pots containing 1 L of mixed quartz sand and vermiculite (1/1). All pots (CRP, CRP + low S, and CRP + high S) were mixed with Cameroonian RP 350 mg P g^−1^ soil (equivalent to P fertilization of 80 kg P ha^−1^). The positive control pots were supplemented with the same amount of P applied as soluble phosphate (KH_2_PO_4_). In all inoculated pots, seedlings were transferred to a pit and finely covered with soil and re-inoculated the following day with 2.5 mL (OD = 0.2 at 620 nm) of the respective bacterial suspension or with 0.05 M sterilized NaCl solution for the control treatments (negative and positive). Pots were watered with 30 mL of the corresponding modified, lacking P, Hoagland solution five times per week and with 30 mL of osmose water twice per week. Salt stress was imposed by adding 20% NaCl to the Hoagland solution until reaching the targeted concentration. Plant growth was documented over six weeks after transplanting in a greenhouse at a day/night temperature 25/23 °C and 75% air humidity. The experimental design was a completely randomized block system with eight treatments and five replications for each treatment. At the harvest, the plant height, number of leaves, and stem diameter were recorded. Then, the aerial part was separated from the root part and plant roots were thoroughly washed in tap water and deionized water. Shoot, root, and total fresh biomass were documented. The dry biomass of shoots and roots was determined after they were oven dried at 60 °C for 72 h. Shoot/root ratio was calculated and the dry matter content was expressed as the percentage of dry weight in fresh weight. Oven-dried tissues were used to determine the shoot and root P concentration by the phospho-vanadomolybdate colorimetric method [20].

### 2.6. Statistical Analyses

All data were analyzed statistically using the analysis of variance test (ANOVA). Mean comparison between treatments was conducted using the Tukey HSD test. Significance was determined at 5% (*p* ≤ 0.05) probability level, and significantly different means were indicated by different letters. All the statistical calculations were performed using SigmaPlot software version 12.3 (Systat Software GmbH, Erkrath, Germany).

## 3. Results

### 3.1. Characterization of Bacterial Strains under Normal Condition

#### 3.1.1. Phosphate Source and Strain-Dependent Phosphate-Solubilizing Efficacy on Agar Plates

**Figure 1:**
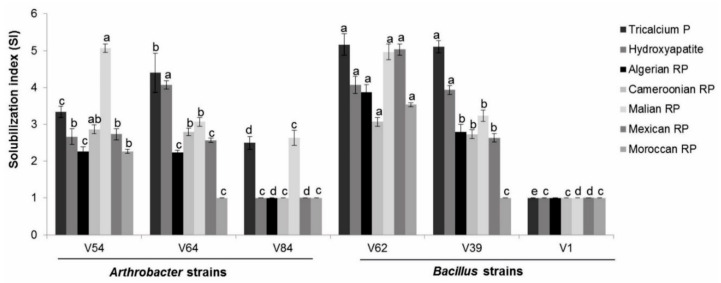
The efficiency of bacterial strains to solubilize inorganic phosphate sources on solid media. The solubilization index of three *Arthrobacter* (V54, V64, and V84) and three *Bacillus* (V62, V39, and V1) strains for the seven different inorganic phosphate sources, as measured on solid agar plates. The results are the means of three replicates. Error bars represent the standard deviation. Different letters indicate significant differences between bacterial strains within the same phosphate source (*p* < 0.05) using the Tukey HSD test. RP = rock phosphate.

Among the six tested bacteria, all strains, except *Bacillus* strain V1, were able to solubilize at least one inorganic phosphate source on the agar plates (Figure 1). The phosphate-solubilizing ability across all P sources: tricalcium phosphate, hydroxyapatite, and Algerian, Cameroonian, Malian, Mexican, and Moroccan rock phosphate (RP) differed depending on the bacterial strain. *Arthrobacter* strain V54 and *Bacillus* strain V62 were the only strains able to solubilize all the seven phosphate sources. *Bacillus* strains V62 and V39 showed the highest solubilization index (SI = 5.1) for tricalcium phosphate (TCP). Hydroxyapatite was best solubilized by V62 and V64 (SI = 4.0). V62 again displayed the highest SI with Algerian RP (3.9), Cameroonian RP (3.0), Mexican RP (5.0), and Moroccan RP (3.5), while V54 with the SI of 5.0 was the most efficient strain in solubilizing Malian RP (Figure 1).

#### 3.1.2. Phosphate Source and Strain-Dependent Phosphate-Solubilizing Efficiency in Liquid Culture under Non-Stress Conditions

**Figure 2:**
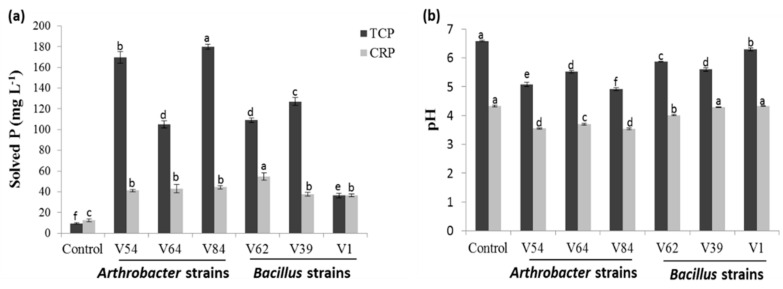
The efficiency of bacterial strains to solubilize inorganic phosphate sources in liquid culture. (**a**) Amount of P (mg L^−1^) released from tricalcium phosphate (TCP) and Cameroonian RP (CRP). (**b**) Accompanying pH changes induced by isolates of National Botanical Research Institute’s Phosphate (NBRIP) medium supplemented with the two phosphate sources without additional salt stress (right hand side). Data are the means of three replicates and error bars represent the standard deviation. The different letters indicate significant difference between bacterial strains for a specific phosphate source (*p* < 0.05) using the Tukey HSD test. Control = NBRIP medium supplied either with TCP or CRP without bacterial inoculation.

To quantify the specific bacterial phosphate-solubilizing activity in liquid culture, we used the most easily solubilized TCP and the local insoluble phosphate, Cameroonian RP (CRP). The amount of soluble P and changes in pH were monitored for five days in NBRIP medium. In the liquid culture supplemented with TCP or CRP, all bacterial strains solubilized phosphate, but at different rates, depending on the strain and the phosphate source. Bacterial inoculation significantly increased the TCP and CRP solubilization compared to the non-inoculated control (Tukey test *p* < 0.05; Figure 2). Bacterial-induced solved P amounts, estimated in the NBRIP supernatant, varied from 36.3 to 179.9 mg L^−1^ in TCP, with the highest solubilization recorded in *Arthrobacter* strains V84 (179.9 mg L^−1^) and V54 (169.6 mg L^−1^), followed by *Bacillus* strain V39 (127.1 mg L^−1^). In contrast, with CRP, the estimated amount of P solubilized varied from 36.4 to 54.5 mg L^−1^ with the maximum concentration in *Bacillus* strain V62 (54.5 mg L^−1^), followed by *Arthrobacter* strains V84 (44.2 mg L^−1^) and V64 (42.9 mg L^−1^). *Bacillus* strain V1, which was unable to show any phosphate-solubilizing activity on solid media supplied with the different phosphate sources, could mobilize phosphate from TCP and CRP in liquid culture. However, compared to all strains, it recorded the lowest P concentration of 36 mg L^−1^ with TCP and CRP. Solubilization of phosphates was associated with a pH decrease in the NBRIP medium (Figure 2). This decrease was observed for all strains with the two phosphate sources. *Arthrobacter* strains were more efficient in decreasing the pH than *Bacillus* strains, regardless of the phosphate source added to the medium. However, the drop in pH in the case of the NBRIP medium supplied with CRP was also observed in the non-inoculated control treatment. The result showed a negative correlation between the soluble P concentration and the pH (*r* = −0.9 and *r* = −0.5; *p* < 0.05) in TCP and CRP, respectively.

### 3.2. Characterization of Bacterial Strains under Salt Stress

#### 3.2.1. Bacillus Strains Tolerated Higher Salt than Arthrobacter Strains and Reveal Best Swarming and Swimming Abilities under Salt Stress Conditions

All the bacterial strains were individually screened for their salt tolerance at graded concentrations of NaCl (2–16%, *w/v*). The results show a higher salt tolerance of the *Bacillus* strains, which even tolerated up to 10% NaCl (except V1), compared to the *Arthrobacter* strains used in this study (Table 1). All bacterial strains except V1 were able to tolerate at least 4% NaCl on agar plates.

Bacterial swarming and swimming abilities differed depending on the strain and the salt (NaCl) concentrations (Table 2). High swarming and swimming abilities were observed in all bacterial strains at 0% NaCl (normal conditions). However, this ability of most bacterial strains decreased with increasing salt concentration. At 2% NaCl, all strains showed at least a low swarming or swimming potential. The *Arthrobacter* strain V54 showed swarming and swimming potential up to 4% NaCl and *Bacillus* strains V62 and V39 even at respectively 10% and 12% NaCl. 

#### 3.2.2. Effect of Phosphate Source and Salt on Phosphate-Solubilizing Activity

**Figure 3:**
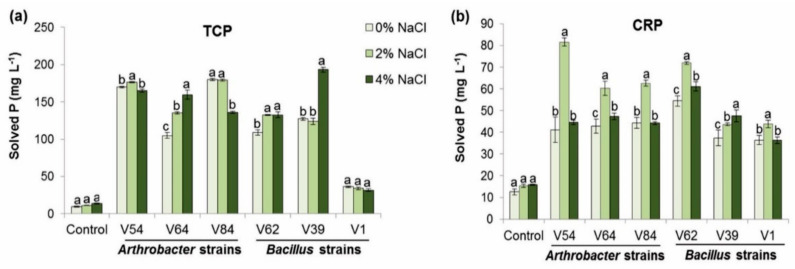
Effect of different NaCl concentrations on phosphate-solubilizing abilities of bacterial strains. (**a**) Amount of P (mg L^−1^) released from tricalcium phosphate (TCP), and (**b**) amount of P (mg L^−1^) released from Cameroonian RP (CRP) by the different bacterial strains at 0%, 2%, and 4% of NaCl. Results are the mean values of three replicates for each treatment. Error bars represent the standard deviation and different letters above the error bars (a,b,c) indicate significant salt effects for each strain separately (*p* < 0.05) using the Tukey HSD test. Control = NBRIP medium supplied either with TCP or CRP without bacterial inoculation.

To determine their phosphate-solubilizing activity under saline conditions, bacterial strains were also tested for their ability to solubilize tricalcium phosphate (TCP) and Cameroonian RP (CRP) in the presence of different concentrations (2% and 4%) of NaCl in NBRIP broth (Table S3). The results were compared to the results obtained under the normal condition (0% of NaCl) to determine the influence of salt on the amount of P released from TCP and CRP in NBRIP medium (Figure 3). Similar to normal conditions, the solubilizing capacity of the strains differ significantly (*p* < 0.05; Tukey HSD test) depending on the phosphate source and the salt concentration. In general, *Arthrobacter* strains were more efficient in solubilizing TCP in the presence of 2% of NaCl, with the highest solubilization in V84 (179.2 mg P L^−1^) and V54 (176.4 mg P L^−1^). In contrast with CRP, the greatest amount of solubilized P at the same concentration of NaCl was found in *Arthrobacter* V54 (81.5 mg P L^−1^) and *Bacillus* strain V62 (71.9 mg P L^−1^). In the presence of 4% NaCl, the highest amount of solubilized P was recorded for *Bacillus* strain V39 (193.1 mg P L^−1^) in TCP, followed by *Arthrobacter* strain V54 (164.9 mg P L^−1^). *Bacillus* strains V62 (61.04 mg P L^−1^) and V39 (47.5 mg P L^−1^) showed the highest capability to release P from CRP (Table S3). Under saline conditions (2% and 4% NaCl), the solubilization of the two phosphate sources in liquid medium corresponded to a decrease in the medium pH (Table S3). This decrease was observed for all strains with the two phosphate sources. *Arthrobacter* strains were more efficient at decreasing the pH than *Bacillus* strains regardless of the phosphate source added to the medium and irrespective of the salt concentration. Soluble P concentrations correlated negatively with the pH of the medium (TCP, *r* = −0.9; CRP, *r* = −0.8, *p* < 0.05) and (TCP, *r* = −0.9; CRP, *r* = −0.7, *p* < 0.05) at 2% and 4% NaCl, respectively.

The analysis of variance also showed a significant effect of salt concentration (*p* < 0.05) on the phosphate-solubilizing activity of strains. Unexpectedly, all bacterial strains expressed a higher phosphate-solubilizing activity of CRP at 2% NaCl compared to normal growth conditions (Figure 3b). *Bacillus* strain V39 showed an even higher phosphate-solubilizing activity when the NaCl concentration increased to 4%. The salt concentration effect varied specifically with the bacterial strain; however, all selected bacterial strains solubilized at least the same amount of P from both phosphate sources (TCP and CRP) under 2% and even 4% NaCl as under normal growth conditions (Figure 3a,b).

### 3.3. Bacterial Inoculations Promoted Tomato Plant Growth and P Uptake Even More Efficiently under Increased Salt Stress and P Deficient Conditions

#### 3.3.1. Verification of Phosphorus Deficiency and Salinity Effects on the Growth and P Uptake of Tomato Plants

**Figure 4:**
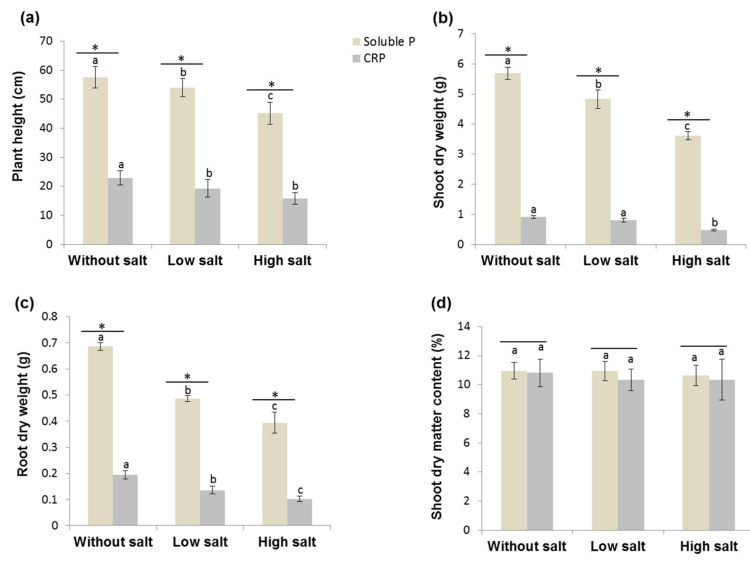
Phosphorus deficiency and salinity affect the growth of tomato plants. Effects of phosphorus stress and salinity on (**a**) plant height, (**b**) shoot, and (**c**) root dry weights, and (**d**) shoot dry matter content of control tomato plants (without bacterial inoculation) grown under different levels of salt stress: without salt (0 ds m^−1^), low salt stress (8 ds m^−1^), and high salt stress (12 ds m^−1^). Peach bars represent plants supplied with soluble phosphate, KH_2_PO_4_, and grey bars plants supplied with hardly accessible phosphate (Cameroonian rock phosphate: CRP). Data shown are the mean of five replications and the error bars represent the standard deviation. Different letters above the error bars (a,b,c) indicate significant difference between salt stress conditions separately for each phosphate fertilization treatment and stars indicate significant difference between plants supplied with soluble phosphate and plants supplied with CRP under the same salt stress condition (*p* < 0.05) using the Tukey HSD test.

To verify the effect of P deficiency and salt stress levels on the growth and P uptake of non-inoculated tomato plants, control plants with added Cameroonian RP and three levels of salt stress were compared to a non-stressed control fertilized with the easily accessible phosphate source (KH_2_PO_4_). The influence of P and salt stress applications on plant growth parameters as well as P uptake were recorded (Figure 4 and Figure S1, Table S4). Salt addition, low salt stress (8 ds m^−1^), and high salt stress (12 ds m^−1^) significantly reduced plant growth, i.e., plant shoot, root, and total dry weights, plant height, number of leaves, and stem diameter, under easily available KH_2_PO_4_ fertilization (Figure 4 and Figure S1). Shoot P concentration increased with increasing salt stress under easily available KH_2_PO_4_ fertilization (Table S4). P stress induced by fertilization with hardly accessible CRP heavily decreased plant growth, i.e., shoot, root, and total dry matter, shoot/root ratio, plant height, number of leaves, and stem diameter, in tomato plants grown regardless of salt stress levels (Figure 4 and Figure S1). Regarding plants supplied with hardly soluble CRP, the tomato plant growth was significantly reduced under high salt stress conditions excluding the number of leaves and stem diameter (Figure 4 and Figure S1). Except for plant height and root dry weight, the combined P deficiency and salt stress effects were not additive for other growth parameters in low salt stress (P stress and low salt stress). The values remained close to that of plants growing under no salt stress (P stress alone). Similarly, the effects of the two factors were not additive for shoot, root, and total P uptake in CRP + high S (Table S4). Generally, the shoot dry matter content was not affected (Figure 4) and shoot/root ratio increased under salt stress regardless of the phosphate treatment (Figure S1).

#### 3.3.2. Selected Bacterial Strains Promoted Tomato Plant Growth Parameters even under Combined P and Salt Stress Conditions

**Figure 5:**
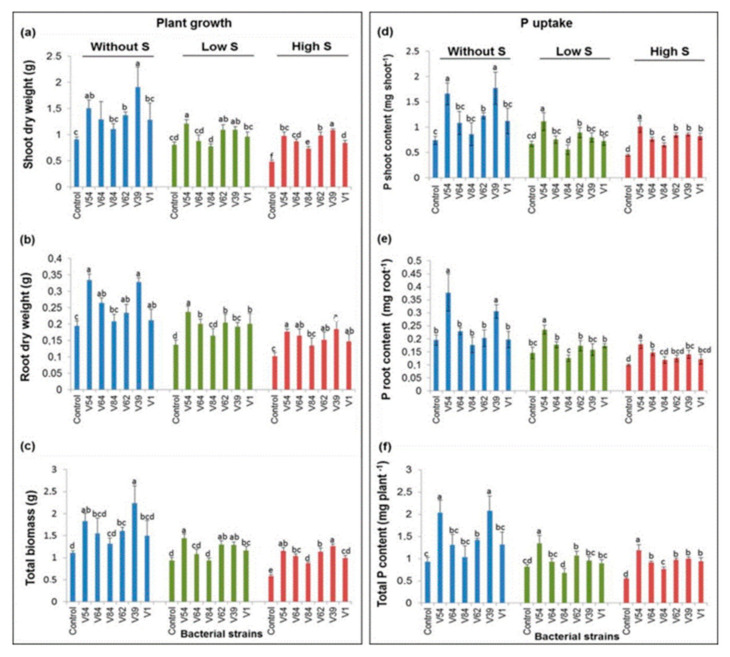
Bacterial inoculations promote tomato plant growth and P uptake under P and salt stress conditions. Effects of bacterial strains compared to the non-inoculated control on plant growth: (**a**) shoot, (**b**) root, (**c**) total biomass, and plant P uptake in (**d**) shoot, (**e**) root, and (**f**) total P contents. Tomato plants grown under P stress and different levels of salt stress were assessed six weeks after planting in a greenhouse. Values are means of five replications and error bars are the standard deviation. The different letters above the error bars indicate significant differences between treatments within a specific stress (*p* < 0.05) using the Tukey HSD test. All plants were under phosphorus stress, amended with hardly accessible Cameroonian RP (CRP). Without S = phosphorus stress without salt stress, Low S = phosphorus stress + low salt stress, and High S = phosphorus stress + high salt stress. Control (non-inoculated), V54, V64, and V84 (*Arthrobacter* sp.), V62, V39, and V1 (*Bacillus* sp.).

All six selected bacterial strains caused a significant increase in shoot dry weight compared to the non-inoculated control plant under P-deficient and high salt stress conditions (CRP + high S, Figure 5a). Among the six bacterial inoculations, two bacterial strains, V54 (*Arthrobacter* sp.) and V39 (*Bacillus* sp.), significantly enhanced (ANOVA, *p* < 0.05) plant height under CRP and CRP + high S; V54 alone induced a significant increase in plant height under CRP + low S. Strain V54 exhibited the greatest effect on plant height under CRP (24.1%) and CRP + low S (26.1%), whereas V39 promoted the highest plant height under CRP + high S (27.8%) (Figure S2).

In the same context, three strains (V39, V54, and V62) led to a significant increase (ANOVA, *p* < 0.05) in the shoot dry weight over the non-inoculated control, respectively, in CRP and CRP + low S. Tomato plants inoculated with *Bacillus* strain V39 showed the highest effect on shoot dry weight in CRP (109.6%) and CRP + high S (122.6%), whereas *Arthrobacter* strain V54 (50.6%) induced the biggest effect on shoot dry weight under CRP + low S. For root dry weight, three bacterial strains (V39, V54, and V64) promoted a significant increase over the negative control under CRP, and all strains, except *Arthrobacter* strain V84, significantly enhanced root dry weight compared to the negative control under CRP + low S and CRP + high S. Plants inoculated with V54 had the maximum effect on root dry weight under CRP (72.1%) and CRP + low S (73.5%), while V39 (80.3%) showed the highest effect on root dry weight under CRP + high S (Figure 5b). With total dry biomass, the significant relative increase in CRP, CRP + low S, and CRP + high S varied between 45% and 103%, 23% and 54%, and 47% and 115%, respectively, compared to the non-inoculated control (Figure 5c). Generally, bacterial inoculation increased the shoot dry matter content, number of leaves, and stem diameter under all stress conditions (Figure S3).

#### 3.3.3. Effect of Bacteria on P Uptake of the Tomato Plant

Phosphorus shoot and root contents as a direct measure of PGP activities were clearly affected by different bacterial inoculants. Significant differences between treatments (ANOVA, *p* < 0.05) were found in the P content of the tomato plants. Three strains (V39, V54, and V62) under CRP, two strains (V54 and V62) under CRP + low S, and all the strains under CRP + high S resulted in significant increases (*p* < 0.05) in P shoot content of tomato plants compared to the negative control (Figure 5d). The maximum increase was recorded for plants inoculated by *Bacillus* strain V39 in CRP (1.8 mg shoot^−1^), while *Arthrobacter* strain V54 induced the highest P shoot content under CRP + low S (1.1 mg shoot^−1^) and CRP + high S (1.0 mg shoot^−1^). Regarding the P root content, inoculation with two bacterial strains (V39 and V54) under CRP, one (V54) under CRP + low S, and three (V39, V54, and V64) under CRP + high S resulted in a significant increase compared to the negative control. *Arthrobacter* strain V54 caused the highest increase under all growth conditions: (0.4 mg root^−1^), (0.2 mg root^−1^), and (0.2 mg root^−1^) respectively under CRP, CRP + low S, and CRP + high S (Figure 5e). Similarly, the result of bacterial inoculation revealed that the highest amount of total P uptake was obtained in tomato plants inoculated with *Bacillus* strain V39 (2.0 mg plant^−1^) and *Arthrobacter* strain V54 (2.0 mg plant^−1^), with an increase of 122% and 118%, respectively, over the non-inoculated control plants under CRP. The maximum value of the total P uptake was achieved by inoculation with *Arthrobacter* strain V54 under CRP + low S (1.3 mg plant^−1^) and CRP + high S (1.2 mg plant^−1^), which induced an increase of 65% and 117%, respectively, over the non-inoculated control (Figure 5f). The observed bacterial-induced increases in plant P uptake were mainly related to increased plant dry matter production. Only the *Arthrobacter* strain V54 additionally induced a significant higher P concentration (mg P per g plant dry matter) in the tomato shoot under all salt conditions (Figure S4) while the *Bacillus* strain V39 inoculation yielded in significant higher shoot dry matter content under all salt conditions compared to the respective non inoculated control (Figure S3).

## 4. Discussion

In this study, we found that selected bacterial strains solubilized P in vitro under normal and saline conditions and in vivo improved tomato plant growth under P stress, and combined P and salt stresses. Bacterial isolates were selected primarily based on their phosphate-solubilizing activity, as well as their salinity tolerance. Notably, in agreement with a previous report [10], our results confirm that bacteria related to *Bacillus* genera reveal high salt tolerance. Most interestingly, the *Bacillus* strain V62 was able to grow at 10% NaCl, while the related strain V39 could develop even under concentrations of up to 14% NaCl. Such bacteria are rarely described in the literature and could be more efficient in saline conditions. Halotolerant bacteria are able to withstand high salt concentrations due to their capability to accumulate compatible osmolytes to maintain intracellular osmotic balance. Indeed these bacteria are crucial for agricultural production and may help plants survive under growth inhibitory levels of salt [21]. The use of halotolerant PGPR is an effective approach that has been employed successfully in various crops to improve their growth and tolerance under salt stress conditions [21]. Our findings especially of the *Bacillus* strain V39 confirm this potential. This strain possibly supported the plant osmotic adjustment under combined P- and salt stresses by accumulating solutes and minerals in the shoot, i.e., increased dry matter content.

Since the motility of bacteria is also an important factor to consider when selecting bacteria as bio-inoculants, it is interesting to note that all bacterial strains tested in this study showed swimming and swarming motility under normal conditions and some, mainly the *Bacillus* strains, even under high salt concentrations. This confirms previous reports indicating the motility of bacterial strains of different genera, including *Arthrobacter* and *Bacillus*, under normal and saline conditions [13,22,23]. Plant colonization is a complex process, and motility of bacteria in soil and/or on plant surfaces is a basic component of this process. Our findings suggest that the bacterial strains investigated here have the potential to effectively colonize the root of the host plant, even under saline conditions, which is the first and foremost step for plant–microbe interactions.

We used an easy to handle and fast solid plate method to check a high number of different rock phosphates or phosphate compounds with selected bacterial strains, since bacterial rock phosphate-solubilizing activity is known to be strain specific and dependent on environmental conditions [11,16]. Strains able to solubilize a wider range of different phosphate compounds, as we demonstrate in our experiments, are likely to be efficient candidates to improve plant growth under P deficiency. The major mechanism of mineral phosphate solubilization in plant-associated bacteria is the production of low-molecular-weight organic acids, which trigger acidification of the soil or media [7,24]. These organic acids can chelate the cation bound to phosphate with their hydroxyl and carboxyl groups [25]. The observed pH decrease in NBRIP medium supplied with TCP from an initial value of 7.0 to a final value of 4.9 indicates that acidification of medium due to organic acid production could facilitate bacterial phosphate solubilization. This is consistent with earlier reports showing that mineral phosphate solubilization is accompanied by a decrease in pH [7]. Surprisingly, in NBRIP medium supplied with CRP, the decrease in pH was also observed in non-inoculated media (the control), suggesting that organic production is not the sole reason for the increase in P concentration in the culture medium, as reported earlier [26]. Physical-chemical processes also contribute to the complex P transfer mechanisms in soils or soil water complexes. An alternative mechanism to organic acid production for solubilization of mineral phosphates is the release of hydrogen ions (H^+^) to the outer surface in exchange for cation uptake, or with the help of H^+^ translocation ATPase [3,24]. For example, assimilation of NH_4_^+^ together with H^+^ excretion supports phosphate solubilization [3]. It emerges from these results that phosphate-solubilizing mechanisms vary, depending on the nature of the phosphate source and the biological processes around. However, the additional application of bacterial strains significantly increased P uptake by the plants compared to application of Cameroonian RP alone.

Although all bacterial strains used in this study were able to solubilize phosphate under salt stress, it was observed that increasing salt concentrations could influence phosphate solubilization either negatively or positively depending on the bacterial strain and the phosphate source. Some bacteria, mainly *Bacillus* strain V39, showed higher phosphate-solubilizing activity with increasing salt concentrations regardless of the phosphate source. Srinivasan and coworkers previously reported that *Aerococcus* and *Pseudomonas* spp. were able to solubilize TCP at different NaCl concentrations [5]. The significant increase in phosphate-solubilizing activity that we observed at 2% NaCl for all tested bacterial strains in the presence of different phosphate sources means that the selected bacterial strains actually require a moderate NaCl concentration for better solubilization. These findings are consistent with previous studies showing that phosphate solubilization by bacteria increased in the presence of salt [8]. Although some bacteria have been reported to show phosphate-solubilizing activity at NaCl concentrations of up to 10% [27], for most of our bacterial strains, we observed that the efficiency of solubilizing the two phosphate sources significantly decreased at 4% NaCl. This decrease can be explained in two ways: (i) salt adversely affects bacterial growth and cell proliferation, resulting in a loss of solubilization efficiency; or (ii) chloride ions (Cl^−^) sequester or neutralize protons or acids produced in the media [8].

In our study, the combined effects of abiotic stresses, i.e., P deficiency and salinity, significantly reduced tomato plant growth, although P stress had a more pronounced impact. This confirms earlier findings that under the combined effects of P and salt stresses, the most growth-limiting factor for barley is P deficiency [2]. Others [28,29] reported similar results in corn and pea also grown under combined nutrient deficiency and salt stress conditions. We observed that the combined effects of P stress and salinity on plant growth and P uptake were most significant under high salt concentrations. Under low salt concentrations, the additive effect of P deficiency and salinity did not always affect plant growth and P uptake. Cultivated tomato is, in any case, a moderately tolerant plant, regulating water and ionic homeostasis [30], and is able to withstand certain concentrations of salt, which is also supported in our experiment. Thus, P deficiency seems to have a stronger impact on tomato plant growth, as confirmed in the present work. Under P starvation, the effect of inoculating tomato plants with *Arthrobacter* and *Bacillus* strains significantly increased shoot and root dry weight by 50.4–109.6% and 9.3–72.1%, respectively, compared to the non-inoculated tomato plant under the same conditions. These values are higher than those of Zhang et al., who found that inoculation of tomato plants by *Acinetobacter* and *Ochracterum* increased shoot dry weight by 26.2–32.6% and root dry weight by 25.6–33.1% compared to non-inoculated plants under P starvation [31]. Our tomato plants inoculated with *Bacillus* strain V39 showed the highest biomass and P uptake increase of 103% and 122%, respectively, compared to the non-inoculated tomato plants supplied with Cameroonian RP without salt stress. This result is in accordance with a recent study showing that *Pantoea* sp. and *Bacillus* sp. contributed better to the plant growth of soybean inoculated with native bacteria isolated from Cameroonian palm tree rhizosphere, in pots supplied with Moroccan RP [32]. Bacterial inoculation with local RP is an economical and sustainable strategy for improving the growth and nutrient accumulation of plants in P-deficient salt-affected soils. It is already established that bacterial inoculation efficiently increases the bioavailability of P in soils fertilized with RP [11,33]. However, our present study has demonstrated for the first time that P availability for tomato plants improved under additional salt stress.

Under high salt stress, our selected bacterial strains even exceeded the plant growth-promoting responses of the P-deficient negative control (without salt stress). The observed positive effects of *Arthrobacter* and *Bacillus* strains on tomato plant growth under combined P and salt stresses are comparable with results from a recent study reporting that, these bacterial strains enhanced plant biomass and P uptake of maize plant under the same stress condition [14]. These results demonstrate that the growth-promoting effects of the here tested bacteria are not dependent on the host plant. Since the inoculated plants were not supplied with any additional source of soluble P, the higher amount of P detected in the shoot or roots of inoculated plants, as well as growth promotion under the different stress conditions may be attributed to the bacterially assisted phosphate solubilization effect from RP. The salt stress tolerance of the plants is perhaps increased by moving the equilibrium of P and other ions in the salt towards P. This clearly demonstrates that bacteria possessing phosphate-solubilizing and salt tolerance abilities, as used in this study, play an important role in promoting plant growth under combined P and salt stresses.

Our findings also suggest that the higher the abiotic stress (P deficiency and salinity), the higher the bacterial effect on plant growth. In fact, some bacterial strains, especially *Arthrobacter* strain V54 and *Bacillus* strains V39, promoted higher growth under double stress conditions in relation to the respective stressed control, compared to P deficiency treatment alone. This could be due to the fact that under increasing combined P and salt stresses, natural adaptation mechanisms of the plant are strongly reduced, and the bacterial effect on the plants becomes stronger. However, the difference between the effects of the single bacterial strains on tomato plant growth, observed in this study, may be attributed to their individual traits and rhizosphere competencies. It is important to select bacterial strains adapted to the environmental conditions where they are later expected to be applied. Our results show that the most efficient bacterial strains possessing various PGP activities, *Arthrobacter* strain V54 and *Bacillus* strainV39, also induced higher and more stable effects in vivo under the different applied stress conditions. Additionally, they seem to act via different mechanisms. *Bacillus* strain V39 induced a higher solute and mineral concentration in tomato shoots with increasing salt stress, which probably supports the plant to mitigate the salt stress [34] while the *Arthrobacter* strain V54 was the only strain increasing both plant growth and additionally P concentration in tomato shoot under all salt concentrations, which indicates a strong P-solubilizing and providing ability. Thus, our study extends the understanding of PGP properties contributed by members of *Arthrobacter* and *Bacillus* genera, and provides important insights into the use of plant–microbe interactions to improve plant growth under combined P and salt stress conditions. Additionally, to the best of our knowledge, the present study is also the first to report the contribution of *Arthrobacter* sp. to the growth of tomato crops under combined P and salt stresses, and the first reporting the positive effect of PGPR on tomato plant growth in vivo through solubilization of RP under saline conditions. Though, the complexity of genes and gene regulation involved in adaption to different stress conditions and to plants is yet still not known and needed.

## 5. Conclusions

For the first time, we demonstrated that *Arthrobacter* and *Bacillus* strains help tomato plants cope better with double stresses of salinity and P deficiency under controlled glasshouse conditions. Our study highlights the capacity of the selected bacteria to solubilize phosphate in the presence of high salt concentrations and to promote tomato plant growth under P deficiency and even under combined P and salt stresses. This provides valuable information for producing effective bio-fertilizers based on the combined application of rock phosphate and halotolerant phosphate-solubilizing bacteria, and offers promising potential to improve plant growth in P-deficient and salt-affected soils. However, since outdoor conditions in agricultural environments are more complex than experimental greenhouse conditions, field studies will be necessary to confirm such findings before these strains can be recommended as bio-fertilizers for commercial applications.

## Figures and Tables

**Table 1:** Salt tolerance of different bacterial strains.

Bacterial Isolates			Salinity Tolerance (% NaCl)							
		0.05	2	4	6	8	10	12	14	16
*Arthrobacter* strains	V54	+	+	+	-	-	-	-	-	-
	V64	+	+	+	-	-	-	-	-	-
	V84	+	+	+	-	-	-	-	-	-
*Bacillus* strains	V62	+	+	+	+	+	+	-	-	-
	V39	+	+	+	+	+	+	+	+	-
	V1	+	+	-	-	-	-	-	-	-

(+) = growth; (-) = no growth.

**Table 2:** Swarming (0.5% agar) and swimming (0.3% agar) abilities of bacterial strains at different concentrations of NaCl.

Bacterial Isolates		0% NaCl		2% NaCl		4% NaCl		8% NaCl		10% NaCl		12% NaCl		14% NaCl		16% NaCl	
		0.5% Agar	0.3% Agar	0.5% Agar	0.3% Agar	0.5% Agar	0.3% Agar	0.5% Agar	0.3% Agar	0.5% Agar	0.3% Agar	0.5% Agar	0.3% Agar	0.5% Agar	0.3% Agar	0.5% Agar	0.3% Agar
*Arthrobacter* strains	V54	+++	+++	+++	+++	++	+++	-	-	-	-	-	-	-	-	-	-
	V64	+++	+++	+++	+++	++	++	-	-	-	-	-	-	-	-	-	-
	V84	+++	+++	++	++	++	++	-	-	-	-	-	-	-	-	-	-
*Bacillus* strains	V62	+++	+++	+++	+++	+++	+++	++	++	++	++	-	-	-	-	-	-
	V39	+++	+++	+++	+++	+++	+++	++	++	++	++	++	++	++	-	-	-
	V1	+++	+++	++	++	-	-	-	-	-	-	-	-	-	-	-	-

(-) = no motility; (++) = low motility; (+++) = high motility.

## Supplementary Materials

The following are available online at https://www.mdpi.com/2076-2607/8/11/1844/s1, Figure S1: Phosphorus deficiency and salinity affects the growth of tomato control plants without bacterial inoculation, Figure S2: Effects of bacterial strains compared to the non-inoculated control on plant height of tomato plants grown under phosphorus stress combined to different level of salt stress, six weeks after planting in greenhouse, Figure S3: Effects of bacterial strains compared to the non-inoculated control on (a) shoot/root dry weight ratio, (b) shoot dry matter content, (c) number of leaves and (d) stem diameter of tomato plants grown under phosphorus stress combined to different level of salt stress, six weeks after planting in greenhouse, Figure S4: Effects of bacterial strains compared to the non-inoculated control on P shoot (a) and P root (b) concentration of tomato plants grown under phosphorus stress combined to different level of salt stress, six weeks after planting in greenhouse, Table S1: Different plant growth-promoting (PGP) potentials of selected bacterial isolates, Table S2: Chemical characteristic of the five inorganic phosphate sources used, Table S3: Efficiency of bacterial strains to solubilize inorganic phosphates at 2% and 4% NaCl in liquid culture: amount of P (mg L^−1^) released from tricalcium P (TCP) and Cameroonian RP (CRP), accompanying pH changes induced by bacterial isolates, Table S4: Effects of P and salt stresses on P shoot and root concentrations and contents, and total P uptake of control tomato plants grown under different salt stress conditions.
