# Supplementary material for: Selected Rhizosphere Bacteria Help Tomato Plants Cope with Combined Phosphorus and Salt Stresses
Source: Microorganisms. 2020 Nov 23;8(11):1844. doi: 10.3390/microorganisms8111844 (PMC7700537; doi:10.3390/microorganisms8111844)
Supplement: Supplementary file 1 [file microorganisms-08-01844-s001.pdf]

Selected rhizosphere bacteria help tomato plants cope with combined phosphorus and salt stresses

Gylaine Vanissa Tchuisseu Tchakounté [1, 2, 3*], Beatrice Berger [4], Sascha Patz [5], Matthias Becker [4], Henri Fankem [2], Victor Désiré Taffouo [2], Silke Ruppel [1]

[1] Leibniz Institute of Vegetable and Ornamental Crops, Theodor-Echtermeyer-Weg 1, 14979 Grossbeeren, Germany

[2] Department of Plant Biology, Faculty of Sciences, University of Douala, 24157 Douala, Cameroon

[3] Faculty of Life sciences Humboldt-University of Berlin, Invalidenstraße 42, 10115 Berlin, Germany

[4] Institute for National and International Plant Health, Julius Kuehn-Institute – Federal Research Centre for Cultivated Plants, Messeweg 11/12, 38104, Braunschweig, Germany

[5] Algorithms in Bioinformatics, Center for Bioinformatics, University of Tuebingen, Sand 14, 72076 Tuebingen, Germany

tchuisseu@igzev.de
beatrice.berger@julius-kuehn.de
sascha.patz@uni-tuebingen.de
matthias.becker@julius-kuehn.de
fankemhenri@yahoo.fr
dtaffouo@yahoo.com
ruppel@igzev.de

[*]correspondence: tchuisseu@igzev.de; Tel: +49 (0)33701-78357

Table S1. Different plant growth-promoting (PGP) potentials of selected bacterial isolates.[1]

Bacterial isolates	Phosphate solubilization							Salinity tolerance				*nif*H Gene
	Tricalcium phosphate	Hydroxy-apatite	Malian RP	Algerian RP	Cameroonian RP	Mexican RP	Moroccan RP	2% NaCl	4% NaCl	6% NaCl	8% NaCl	
V54	+	+	+	+	+	+	+	+	+	-	-	-
V64	+	+	+	+	+	+	-	+	+	-	-	+
V84	+	-	+	-	-	-	-	+	+	-	-	-
V62	+	+	+	+	+	+	+	+	+	+	+	-
V39	+	+	+	+	+	+	-	+	+	+	+	+
V1	-	-	-	-	-	-	-	+	-	-	-	-

(+) = positive, (-) = negative

1. Tchuisseu Tchakounté, G.V.; Berger, B.; Patz, S.; Fankem, H.; Ruppel, S. Community structure and plant growth-promoting potential of cultivable bacteria isolated from Cameroon soil. *Microbiological Research* **2018**, *214*, 47-59, doi:https://doi.org/10.1016/j.micres.2018.05.008.

Table S2. Chemical characteristic of the five inorganic phosphate sources used.

**Origin**	**(%)**								**mg kg^{-1}**		
	Total P_2O_5	Available P	K	Ca	Mg	Na	Fe	Al	Mn	Zn	Cu
**Algeria**	13.85	Nd	0.12	Nd	0.78	1.08	0.27	0.24	Nd	97.50	5.72
**Cameroon**	11.15	Nd	0.04	Nd	0.08	0.006	30.60	3.34	Nd	46.2	10.2
**Mali**	30.00	12.98	0.06	28.19	0.13	0.23	3.84	0.80	8360	87	51
**Mexico**	28.00	8.87	0.22	25.94	0.22	0.36	0.44	0.58	788	103	18
**Morocco**	13.00	9.33	0.09	28.83	1.93	0.55	0.27	0.42	96	219	38

Nd: not determined

Table S3. Efficiency of bacterial strains to solubilize inorganic phosphates at 2 % and 4 % NaCl in liquid culture: amount of P (mg L^{-1}) released from tricalcium P (TCP) and Cameroonian RP (CRP), accompanying pH changes induced by bacterial isolates.

Bacterial strains	2 % NaCl				4 % NaCl			
	TCP		CRP		TCP		CRP	
	Amount of released P (mg L^{-1})	pH	Amount of released P (mg L^{-1})	pH	Amount of released P (mg L^{-1})	pH	Amount of released P (mg L^{-1})	pH
Control	11.2 ± 0.30 ^{f}	6.7 ± 0.01 ^{a}	15.4 ± 0.70 ^{f}	4.5 ± 0.23 ^{a}	13.76 ± 0.53 ^{e}	6.6 ± 0.005 ^{a}	15.8± 0.30 ^{d}	4.5 ± 0.02 ^{a}
V54	176.4 ± 0.48 ^{a}	5.2 ± 0.01 ^{f}	81.5 ± 1.86 ^{a}	3.7 ± 0.02 ^{f}	164.9 ± 2.22 ^{b}	5.4 ± 0.02 ^{ef}	44.5 ± 1.03 ^{b}	4.1 ± 0.01 ^{c}
V64	135.2 ± 1.62 ^{bc}	5.6 ± 0.02 ^{d}	60.3 ± 3.17 ^{c}	3.8 ± 0.005 ^{ef}	159.5 ± 6.03 ^{b}	5.5 ± 0.01 ^{d}	47.2 ± 1.47 ^{b}	4.1 ± 0.02 ^{c}
V84	179.2 ± 1.16 ^{a}	5.2 ± 0.02 ^{e}	62.6 ± 1.28 ^{c}	3.8 ± 0.07 ^{e}	136.0 ± 1.54 ^{c}	5.4 ± 0.04 ^{f}	44.3 ± 0.60 ^{b}	3.9 ± 0.01 ^{d}
V62	132.5 ± 0.79 ^{c}	5.9 ± 0.01^{c}	71.9 ± 0.62 ^{b}	4.1 ± 0.02 ^{d}	132.6 ± 3.71 ^{c}	5.8 ± 0.01 ^{c}	61.0 ± 2.12 ^{a}	4.0 ± 0.1 ^{c}
V39	123.7 ± 4.37 ^{d}	5.9 ± 0.005 ^{c}	43.6 ± 0.62 ^{e}	4.5 ± 0.01 ^{b}	193.1 ± 3.16 ^{a}	5.5 ± 0.01 ^{de}	47.6 ± 2.67 ^{b}	4.3 ± 0.01 ^{b}
V1	34.2 ± 1.89 ^{e}	6.5 ± 0.02 ^{d}	43.7 ± 1.68 ^{d}	4.4 ± 0.01 ^{c}	31.9 ± 1.72 ^{d}	6.4 ± 0.01 ^{b}	36.3 ± 1.54 ^{c}	4.3 ± 0.01 ^{b}

Results are the mean values of three replicates for each treatment and the error bars represent the standard deviation. The different letters indicate significant differences between bacterial strains separately for each treatment (NaCl concentration and phosphate source) ($p < 0.05$) using the Tukey HSD test. TCP = tricalcium phosphate and CRP = Cameroonian RP; Control = National Botanical Research Institute’s Phosphate (NBRIP) medium supplied either with TCP or CRP without bacterial inoculation; V54, V64, and V84 = *Arthrobacter* strains and V62, V39, and V1= *Bacillus* strains.

Table S4. Effects of P and salt stresses on P shoot and root concentrations and contents, and total P uptake of control tomato plants grown under different salt stress conditions.

Plant organs		Growth conditions	Soluble P	Cameroonian RP (CRP)
**P concentration**	P Shoot (mg g^{-1})	Without salt	8.29 ± 0.66 ^{a}	0.81 ± 0.05 ^{a}
		Low salt	11.03 ± 0.47 ^{b}	0.84 ± 0.10 ^{a}
		High salt	12.14 ± 0.90 ^{c}	0.92 ± 0.08 ^{a}
	P Root (mg g-1)	Without salt	4.06 ± 0.47 ^{b}	1.00 ± 0.06 ^{a}
		Low salt	5.26 ± 0.26 ^{a}	1.07 ± 0.13 ^{a}
		High salt	4.54 ± 0.54 ^{b}	0.99 ± 0.10 ^{a}
**P content**	P Shoot (mg $shoot^{-1}$)	Without salt	55.2 ± 1.62 ^{a}	0.7 ± 0.08 ^{a}
		Low salt	53.2 ± 2.61 ^{a}	0.7 ± 0.04 ^{a}
		High salt	43.8 ± 1.82 ^{b}	0.4 ± 0.02 ^{a}
	P Root (mg P $root^{-1}$)	Without salt	2.8 ± 0.35 ^{a}	0.2 ± 0.01 ^{a}
		Low salt	2.7 ± 0.24 ^{a}	0.1 ± 0.02 ^{a}
		High salt	1.8 ± 0.10 ^{b}	0.1 ± 0.002 ^{a}
**Total P uptake**	Total P (mg P $plant^{-1}$)	Without salt	57.9 ± 1.36 ^{a}	0.9 ± 0.09 ^{a}
		Low salt	55.9 ± 2.67 ^{a}	0.8 ± 0.04 ^{a}
		High salt	45.5 ± 1.85 ^{b}	0.5 ± 0.02 ^{a}

Without salt = (0 ds m^{-1}), low salt stress = (8 ds m^{-1}) and high salt stress = (12 ds m^{-1}). Data are means of five replicates for each treatment. Error represent the standards deviation and different letters indicate significant difference between salt stress conditions within the same phosphate fertilization ($p<0.05$) using the Tukey HSD test.

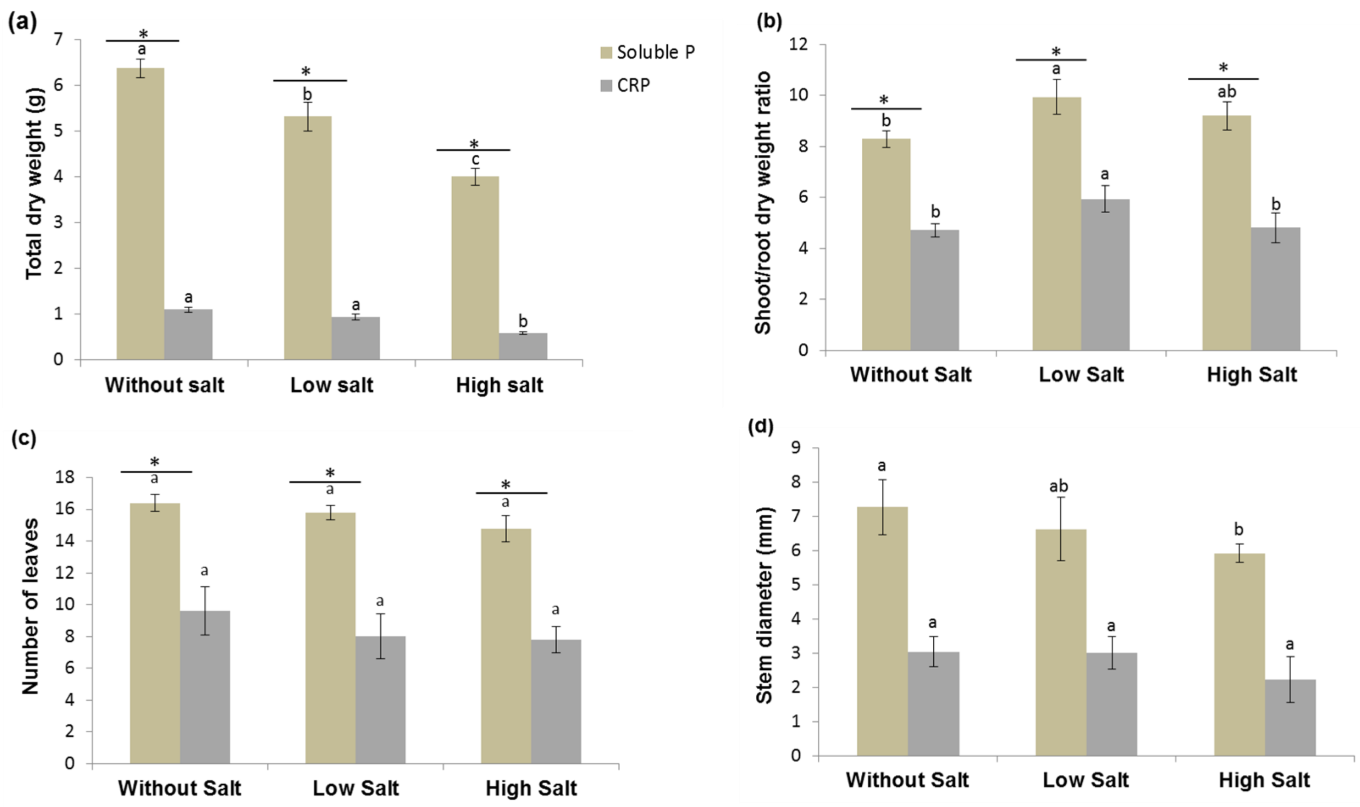

Figure S1. Phosphorus deficiency and salinity affect the growth of tomato control plants without bacterial inoculation. Effects of phosphorus stress and salinity on (**a**) total biomass, (**b**) shoot/root dry weight ratio, (**c**) number of leaves, and (**d**) stem diameter of control tomato plants (without bacterial inoculation) grown under different levels of salt stress: without salt (0 ds m^{-1}), low salt stress (8 ds m^{-1}) and high salt stress (12 ds m^{-1}). Peach bars represent plants supplied with soluble phosphate, KH_2PO_4, and grey bars plants supplied with hardly accessible phosphate (Cameroonian rock phosphate: CRP). Data shown are the mean of five replications and the error bars represent the standard deviation. Different letters above the error bars (a,b,c) indicate significant difference between salt stress conditions separately for each phosphate fertilization treatment and stars indicate significant difference between plants supplied with soluble phosphate and plants supplied with CRP under the same salt stress condition ($p < 0.05$) using the Tukey HSD test.

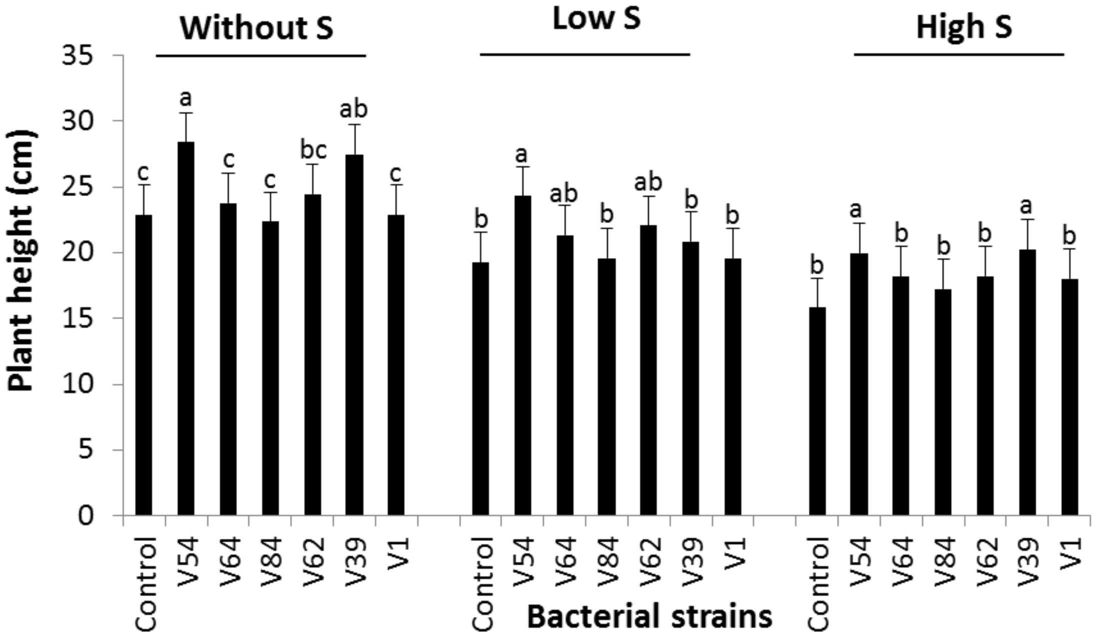

Figure S2. Effects of bacterial strains compared to the non-inoculated control on plant height of tomato plants grown under phosphorus stress combined to different level of salt stress, six weeks after planting in greenhouse. Data are means of five replicates for each treatment. Error represent the standards deviation and different letters indicate significant difference between salt stress conditions within the same phosphate fertilization ($p<0.05$) using the Tukey HSD test. All plants were under phosphorus stress, amended with hardly accessible Cameroonian RP (CRP). Without S = phosphorus stress without salt stress, Low S = phosphorus stress + low salt stress, and High S = phosphorus stress + high salt stress. Control (non-inoculated), V54, V64 and V84 (*Arthrobacter* sp.), V62, V39 and V1 (*Bacillus* sp.).

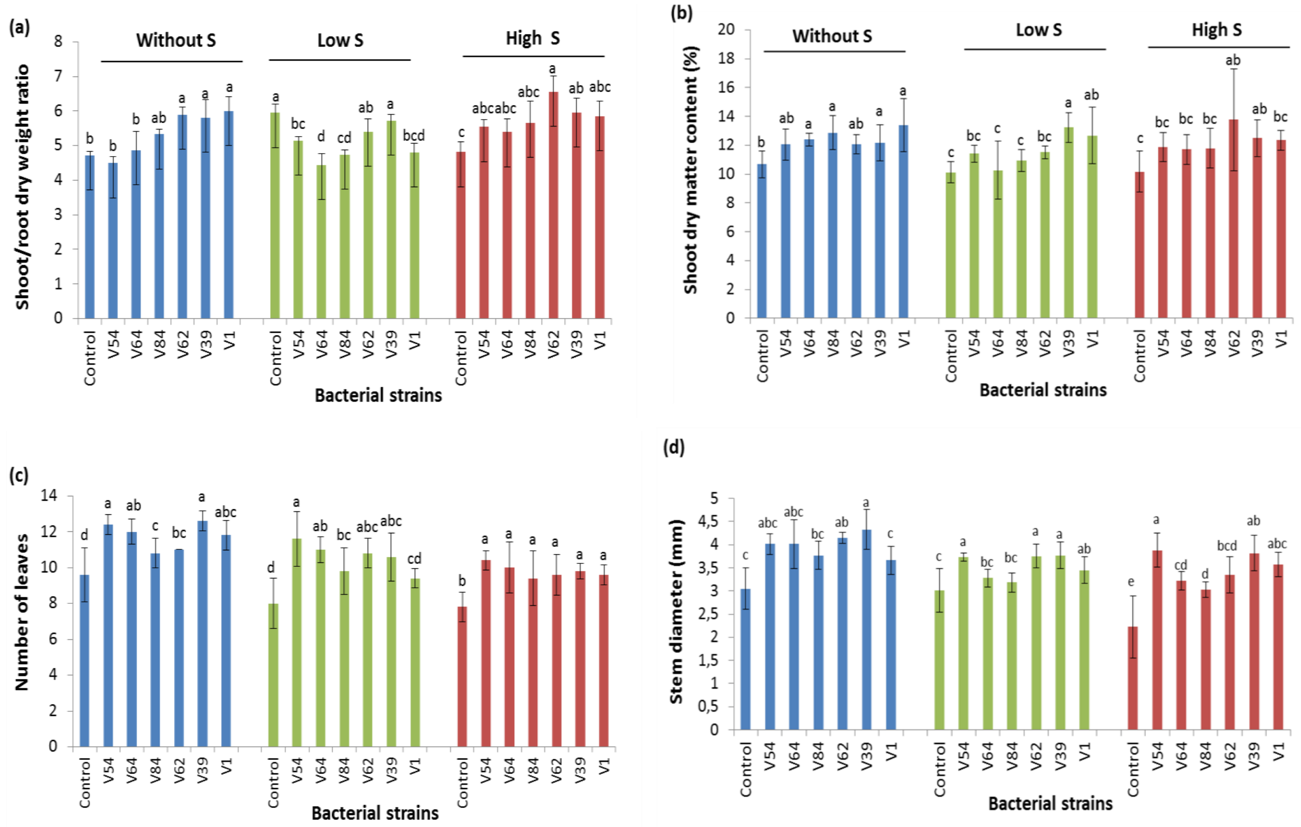

Figure S3. Effects of bacterial strains compared to the non-inoculated control on (a) shoot/ root dry weight ratio, (b) shoot dry matter content, (c) number of leaves and (d) stem diameter of tomato plants grown under phosphorus stress combined to different level of salt stress, six weeks after planting in greenhouse. Data are means of five replicates for each treatment. Error represent the standards deviation and different letters above the error bars indicate significant difference between salt stress conditions within the same phosphate fertilization ($p<0.05$) using the Tukey HSD test. All plants were under phosphorus stress, amended with hardly accessible Cameroonian RP (CRP). Without S = phosphorus stress without salt stress, Low S = phosphorus stress + low salt stress, and High S = phosphorus stress + high salt stress. Control (non-inoculated), V54, V64 and V84 (*Arthrobacter* sp.), V62, V39 and V1 (*Bacillus* sp.).

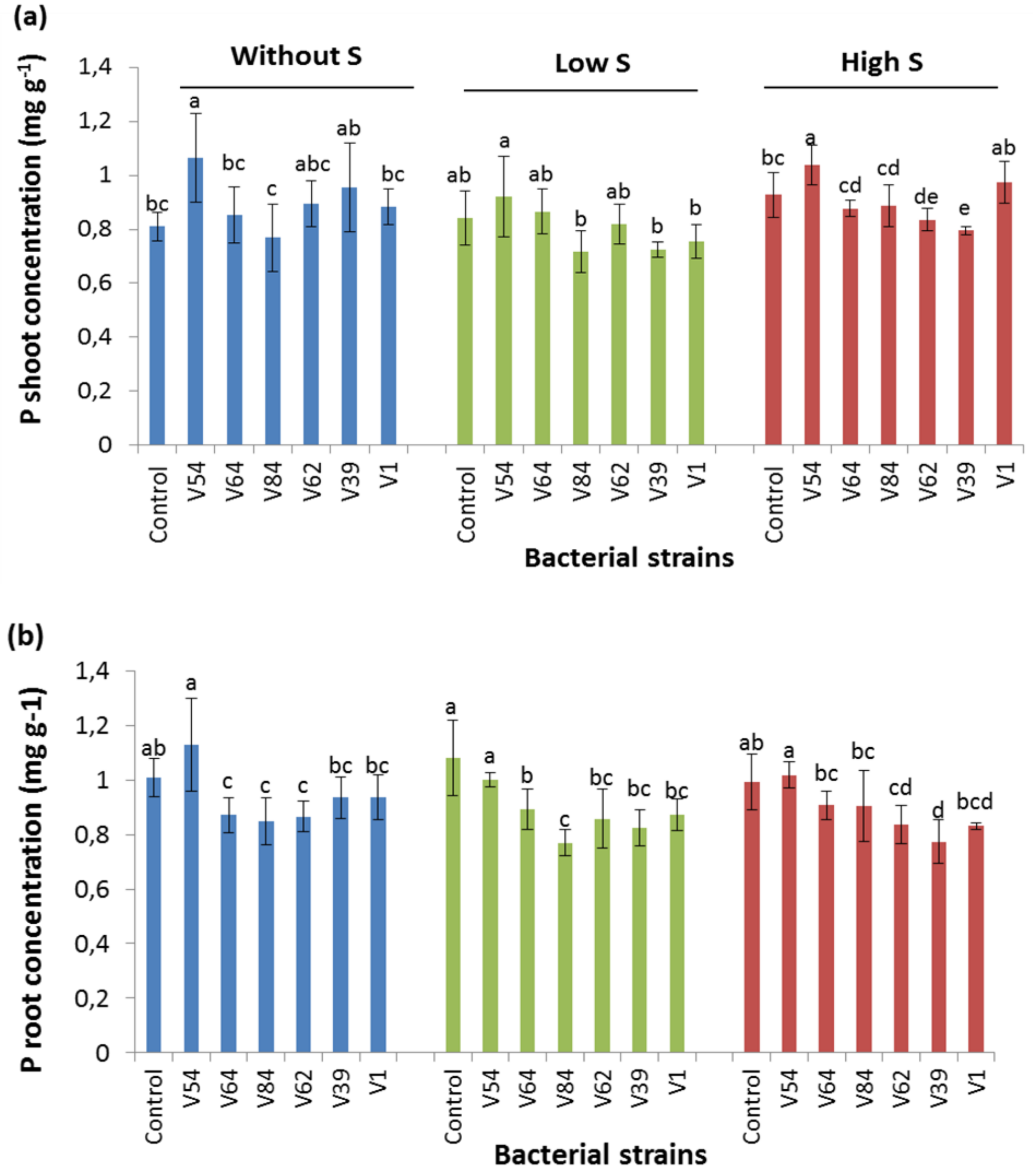

Figure S4. Effects of bacterial strains compared to the non-inoculated control on P shoot (a) and P root (b) concentration of tomato plants grown under phosphorus stress combined to different level of salt stress, six weeks after planting in greenhouse. Data are means of five replicates for each treatment. Error represent the standards deviation and different letters above the errors bars indicate significant difference between salt stress conditions within the same phosphate fertilization ($p<0.05$) using the Tukey HSD test. All plants were under phosphorus stress, amended with hardly accessible Cameroonian RP (CRP). Without S = phosphorus stress without salt stress, Low S = phosphorus stress + low salt stress, and High S = phosphorus stress + high salt stress. Control (non-inoculated), V54, V64 and V84 (*Arthrobacter* sp.), V62, V39 and V1 (*Bacillus* sp.).
